# Computational genes: a tool for molecular diagnosis and therapy of aberrant mutational phenotype

**DOI:** 10.1186/1471-2105-8-365

**Published:** 2007-09-28

**Authors:** Israel M Martínez-Pérez, Gong Zhang, Zoya Ignatova, Karl-Heinz Zimmermann

**Affiliations:** 1 Institute of Computer Technology, Hamburg University of Technology, Hamburg 21073, Germany; 2 Cellular Biochemistry, Max Planck Institute for Biochemistry, Martinsried 82152, Germany

## Abstract

**Background:**

A finite state machine manipulating information-carrying DNA strands can be used to perform autonomous molecular-scale computations at the cellular level.

**Results:**

We propose a new finite state machine able to detect and correct aberrant molecular phenotype given by mutated genetic transcripts. The aberrant mutations trigger a cascade reaction: specific molecular markers as input are released and induce a spontaneous self-assembly of a wild type protein or peptide, while the mutational disease phenotype is silenced. We experimentally demostrated in *in vitro *translation system that a viable protein can be autonomously assembled.

**Conclusion:**

Our work demostrates the basic principles of computational genes and particularly, their potential to detect mutations, and as a response thereafter administer an output that suppresses the aberrant disease phenotype and/or restores the lost physiological function.

## Background

Finite state automata operating at molecular scale [1-6] can conceptually be used for applications in the living cell. Head *et al*. [1] proposed an *in vivo *biomolecular model in which computations manipulate a plasmid DNA. The solution of the problem is given by either the longest or shortest plasmid at the end of the computation. Henkel *et al*. [2] implemented a similar *in vivo *mechanism in which computations are conducted on a DNA sequence constituting of an open reading frame controlled through a strong promoter. Sequential plasmid manipulation finds a final plasmid construct containing the computational solution allowing the *in vivo *transcription and translation into a protein. The first autonomous finite state automaton was proposed in [3-5]. It is a 2-state 2-symbol automaton composed of DNA strands and enzymes. Benenson *et al*. [6] used this automaton to carry out *in vitro *molecular diagnosis and therapy. For this, an anti-sense drug (oligonucleotide – a short single stranded DNA molecule) is released if certain diagnostic conditions are true, i.e., low expression levels of certain mRNAs and high expression levels of others. The diagnostic conditions can be viewed as transitional steps in a finite state automaton. If the automaton reached a final state, indicating that all conditions are met, then the drug enclosed in the loop of a hairpin shaped oligonucleotide is released. This *if-then *mechanism is a new element of autonomous molecular computation. Unfortunately, the proposed mechanisms would not work in a living cell as unwanted side effects raised by supporting molecules (particularly the *Fok*I enzyme carrying out the transitions) would be a major problem [7,8]. Moreover, this molecular mechanism is limited in its ability to logically control gene expression as it allows for administering as output only small molecules.

Nevertheless, this work is an important conceptual step forward to link molecular automata to molecular diagnosis and therapy. The first autonomous finite state machine working in the living cell of *E. coli *was proposed by Nakagawa *et al*. [9]. This approach is based on a length encoding automaton model [10] whose input string is encoded by an mRNA molecule. The computation of this mRNA molecule is accomplished by the biosynthesis mechanism of the cell combined with four-base codon techniques [11,12] yielding a protein of interest.

## Results and discussion

### Computational genes

**Figure 1:**
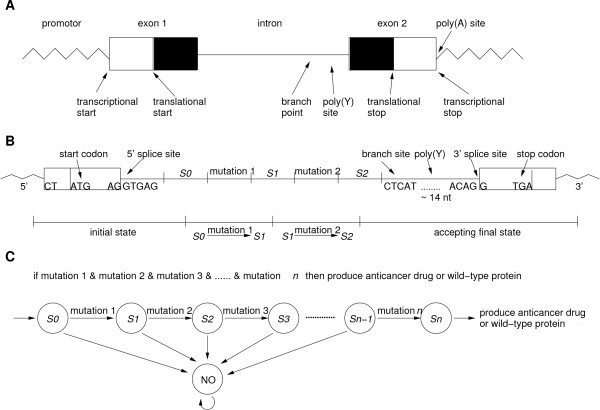
**Design of computational gene**. A) Most eukaryotic genes are organized as alternating sequences of coding (exons) and non-coding (introns) segments. Conserved regions in the introns, e.g., pyrimidine rich region (poly(Y)), 5'-splice junction (AG/GTGAG), AG dinucleotide at the 3'-splice junction, and branch point sequence (CTCAT) guarantee proper splicing. These conserved regions are maintained in the structural moiety of the computational gene. B) Schematic representation of self-assembled (functional) gene encoding diagnostic rule (2), for *n *= 2. The initial state comprises promoter, first exon, and 5'-splicing site, the transition rules are placed in the intron region, and the final state includes branch site, poly(Y)-region, 3'-splice site, and second exon. C) Finite state automaton implementing diagnostic rule (2). The automaton starts in the initial state *S*_0 _and transits into the final state *S*_*n *_if all mutations are present.

Here we present a new molecular automaton, called computational gene, consisting of a structural and functional moiety which is designed such that it might work in a cellular environment. The structural part is a naturally occurring gene, which is used as a skeleton to encode the input and the transitions of the automaton (Figure 1A). The conserved features of a structural gene (e.g., DNA polymerase binding site, start and stop codons, and splicing sites) serve as constants of the computational gene, while the coding regions, the number of exons and introns, the position of start and stop codon, and the automata theoretical variables (symbols, states, and transitions) are the design parameters of the computational gene. The constants and the design parameters are linked by several logical and biochemical constraints (e.g., encoded automata theoretic variables must not be recognized as splicing junctions). The input of the automaton are molecular markers given by single stranded DNA (ssDNA) molecules. These markers are signalling aberrant (e.g., carcinogenic) molecular phenotype [13] and turn on the self-assembly of the functional gene. If the input is accepted, the output encodes a double stranded DNA (dsDNA) molecule, a functional gene which should be successfully integrated into the cellular transcription and translation machinery producing a wild type protein or an anti-drug (e.g., short peptide) (Figure 1B). Otherwise, a rejected input will assemble into a partially dsDNA molecule which cannot be translated. A computational gene can be described by a finite automaton *M *such that the set of strings accepted by *M *(language) is given by those dsDNA molecules that a translation system recognises as genes. To this end, observe that linear self-assembly is equivalent to regular languages [14], and regular languages are exactly those languages that are accepted by finite automata. Therefore, we can expect to construct a functional gene based only on the linear self-assembly of oligonucleotides or duplex DNA with sticky ends, controlled by a finite state machine. The structural gene resembles the natural structure of the genetic information in the cell and bears all the regulatory elements allowing the functional gene to be successfully translated into a protein. As there is no size restriction on the structural gene, there would be also no size limitation for the functional gene.

### Diagnosis and therapy

We study the hypothetical application of simple molecular computations to correct an aberrant mutation in a gene that can trigger a disease-phenotype. One of the most prominent examples is the tumor suppressor p53 gene, which is present in every cell, and acts as a guard to control the growth. Mutations in this gene can abolish its function, allowing uncontrolled growth that can lead to cancer [15,16].

A single disease-related mutation can be diagnosed and treated by the following diagnostic rule,

if proteinX_mutated_at_codon_Ythen produce_drug fi
 MathType@MTEF@5@5@+=feaafiart1ev1aaatCvAUfKttLearuWrP9MDH5MBPbIqV92AaeXatLxBI9gBaebbnrfifHhDYfgasaacH8akY=wiFfYdH8Gipec8Eeeu0xXdbba9frFj0=OqFfea0dXdd9vqai=hGuQ8kuc9pgc9s8qqaq=dirpe0xb9q8qiLsFr0=vr0=vr0dc8meaabaqaciaacaGaaeqabaqabeGadaaakeaafaqaaeGabaaabaGaeeyAaKMaeeOzayMaeeiiaaIaeeiCaaNaeeOCaiNaee4Ba8MaeeiDaqNaeeyzauMaeeyAaKMaeeOBa4gcbiGae8hwaGLaee4xa8LaeeyBa0MaeeyDauNaeeiDaqNaeeyyaeMaeeiDaqNaeeyzauMaeeizaqMaee4xa8LaeeyyaeMaeeiDaqNaee4xa8Laee4yamMaee4Ba8MaeeizaqMaee4Ba8MaeeOBa4Maee4xa8Lae8xwaKfabaGaeeiDaqNaeeiAaGMaeeyzauMaeeOBa4MaeeiiaaIaeeiCaaNaeeOCaiNaee4Ba8MaeeizaqMaeeyDauNaee4yamMaeeyzauMaee4xa8LaeeizaqMaeeOCaiNaeeyDauNaee4zaCMaeeiiaaIaeeOzayMaeeyAaKgaaaaa@6E91@

**Figure 2:**
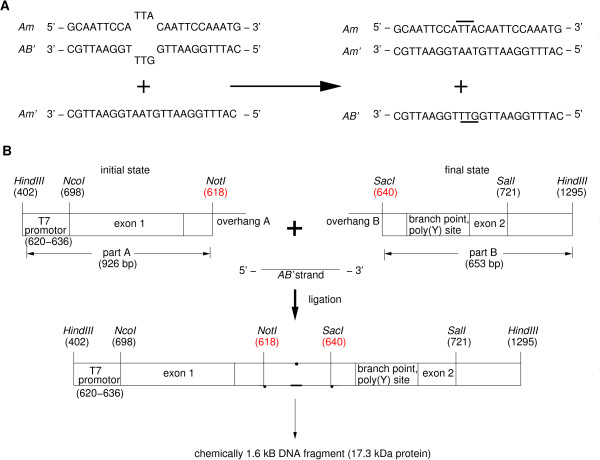
**Design and assembly of the computational gene exemplified with one eukaryotic gene**. A) Diagnosis. The 24 nt long double-stranded diagnostic duplex *Am/AB' *bears three mismatches positioned at the centre. The single-strand *Am *represents the mutation signal and the *AB' *strand the diagnostic signal. The displacement of *AB' *from *Am *is thermodynamically favourable due to the full complementary of *Am' *to *Am*. Regions highlighted in bold show the positions where mismatches were located before strand displacement. B) Therapy. The eukaryotic hID1 gene is the skeleton of the computational gene. The conserved regions of hID1 serve as constants for designing a functional gene being (for simplicity and demonstration purposes) a part of the hID1 gene product itself. For this reason, the intron sequence of hID1 was modified to construct the required initial and final accepting states, keeping the conserved splicing signals intact. The three elements, e.g., initial state (part A), diagnostic signal (*AB'*), and final state (part B), revealed a functional 1.6 kb gene encoding a 155 amino acid long (17.3 kDa) protein.

This rule could allow a protein with a pathogenic mutation to execute its natural physiological function. For instance, a mutation at codon 249 in the p53 protein is characteristic for hepatocellular cancer [15,16] and the CDB3 peptide (nine amino acids) binds to the p53 core domain and stabilises its fold [17]. Although restoring the tumor suppressor activity of the p53 mutants with small stabilising molecules is a promising strategy in cancer therapy, different classes of mutations will require different rescue strategies [18]. The rule (1) can be implemented by a two-state one-symbol automaton consisting of two partially dsDNA molecules and one ssDNA molecule (symbol), which corresponds to the disease-related mutation and provides a molecular switch for the linear self-assembly of the functional gene (Figure 2B, [see Additional file 1]).

To process diagnostic rule (1), the molecular automaton must be able to detect point mutations. This task is based on a diagnostic complex, a dsDNA molecule consisting of a single-stranded mutation signal and a single-stranded diagnostic signal (Figure 2A, [see Additional file 2]). Both strands imperfectly pair in the region that resembles an aberrant mutation to be detected. An mRNA molecule bearing a mutation (e.g., oncogenic) will trigger the dissociation of the diagnostic complex and will pair to the mutation signal. The latter process is thermodynamically driven by the higher stability of the DNA/RNA duplex over the mismatched DNA/DNA diagnostic complex and favourable due to its increased complementarity [19,20].

Moreover, the resulting DNA/RNA hybrid complex will act as a substrate for the cellular RNase H, destroying the RNA component of the duplex [21]. The released single-stranded diagnostic signal links the assembly of the functional gene (Figure 2B, [see Additional file 2]), whose structure is completed by cellular ligase present in both eukaryotic and prokaryotic cells. The transcription and translation machinery of the cell is then in charge of therapy and administers either a wild-type protein or an anti-drug. In both cases, the pathogenic phenotype is suppressed, either by a replacement with the wild-type protein or by a release of a small molecule that may stabilise the aberrant mutant protein, and thus providing the physiological functionality of the wild-type. This idea of favouring the full over the partial complementarity of the base pairing was implemented in robust DNA-based machines 'fuelled' by the DNA/DNA-cross-pairing [22-24]. Although mechanistically simple and quite robust on molecular level, several issues need to be addressed before an *in vivo *implementation of computational genes can be considered. First, the DNA material must be internalised into the cell, specifically into the nucleus. The transfer of DNA or RNA through biological membranes is a key step in the drug delivery [25]. Nuclear localisation signals can be irreversibly linked to one end of oligonucleotides, forming an oligonucleotide-peptide conjugate that allows effective internalisation of DNA into the nucleus [26,27]. In addition, the DNA complexes should have low immunogenicity to guarantee their integrity in the cell and their resistance to cellular nucleases. Current strategies to eliminate nuclease sensitivity include modifications of the oligonucleotide backbone such as methylphosphonate [28] and phosphorothioate (S-ODN) oligodeoxynucleotides [29], but along with their increased stability, modified oligonucleotides often have altered pharmacologic properties [30,31]. Finally, similar to any other drug, DNA complexes could cause nonspecific and toxic side effects. *In vivo *applications of antisense oligonucleotides showed that toxicity is largely due to impurities in the oligonucleotide preparation and lack of specifity of the particular sequence used [30-32]. Undoubtedly, progress on antisense biotechnology will also result in a direct benefit to the model of computational genes.

### Implementation

To test the feasibility of the principle of computational genes, we developed an *in vitro *system making use of the following assumptions: (i) diagnostic and therapy reactions are simulated in one-single step process; (ii) diagnostic complexes contain three adjacent mismatches representing a whole codon replacement (single amino-acid point mutation); (iii) RNA molecules are replaced by ssDNA during simulations.

### Diagnostic complexes

Clearly, the diagnostic complexes must be thermodynamically stable in the cell in order to avoid misactivations (false positives) of functional genes. For this, we conducted strand exchange experiments with single-stranded and double-stranded DNA to determine the strand displacement conditions in *in vitro *experiments [see Additional file 3]. The single strand *Am*' mimics the mutated 'cancerous' mRNA sequence and its addition to the diagnostic complex *Am/AB' *triggers the release of the diagnostic signal *AB'*, and the increase in the *Am' *concentration enhanced the strand displacement rate (Table 1). The pairing of *Am' *with the mutation signal *Am *is thermodynamically favorable due to the new complementary base pairs added in the *Am/Am' *duplex and increased stability of the complex at physiological temperature: melting temperature *T*_*m *_= 60°C in buffer and 62°C in buffer containing crowding agent mimicking the cellular environment [33]; for comparison the *T*_*m *_of *Am/AB' *duplex is 51°C in buffer and 53°C in crowded conditions. The crowding environment did not influence the melting temperature of the duplexes. The diagnostic complexes were stable at physiological temperature and only 13.1% of the diagnostic signals (*AB'*) underwent spontaneous release [see Additional file 4]. The results suggest that the perfect matching is a favorable state and based on this we can conclude a displacement of the diagnostic signal by the mutant mRNA. The stability of the diagnostic complexes can be further optimised by designing diagnostic complexes comprising longer sequences, which will increase melting temperature and reduce spontaneous disassembly. The latter will decrease the displacement rate and the dissociation of a duplex longer than 10–12 bp will take an enormous amount of time  *in vivo*. Therefore, an optimal design of a diagnostic complex should be based on consideration of those three variables.

**Table 1:** Rate-constants of the strand-exchange reactions in phosphate buffer

Ratio [*Am/AB'*]: [*Am'*]	*k *(10^-2^sec^-1^)	Correlation coefficient
1: 0.25	1.258 ± 0.049	0.99689
1: 0.50	2.325 ± 0.029	0.99985
1: 0.75	3.325 ± 0.047	0.99956
1: 0.85	3.846 ± 0.038	0.99871
1: 1	4.476 ± 0.022	0.99996
1: 2	4.524 ± 0.054	0.99945

#### *In vitro *translation

**Figure 3:**
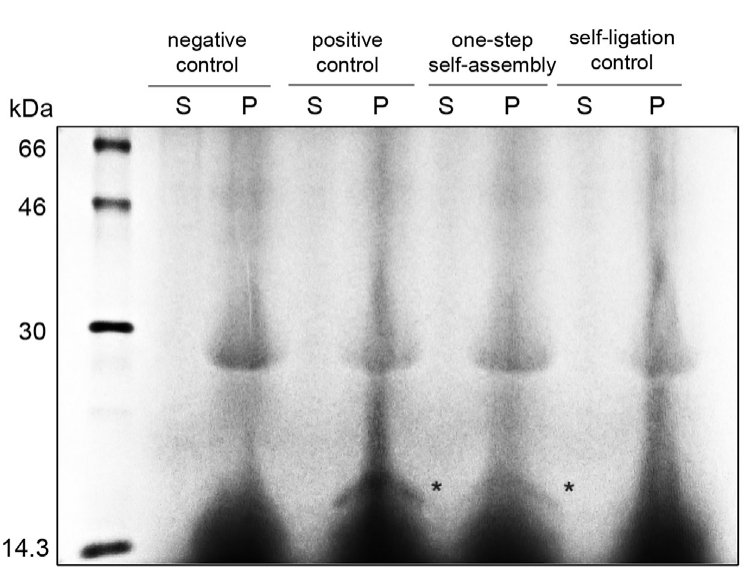
**One-step assembly of the functional gene in eukaryotic *in vitro *translation system**. The mutated DNA, the diagnostic complex and the non-assembled gene components were mixed in equimolar ratio and added to the *in vitro *translation reaction (lane: one step self-assembly). The translation product with a molecular mass of 17.3 kDa is marked by asterisk. Reaction containing only the intact hID1 gene served as positive control (lane: positive control); reaction lacking the mutated DNA was used to test the self-ligation of the diagnostic complex (lane: self-ligation control). The low concentration of the output product severely restricted the detection techniques that can be used to detect the translation product, i.e., in this case only by radioactivity. Note that the eukaryotic transcription/translation coupled system gives two high background signals at 10–13 and 28 kDa. The background that arises from the *in vitro *translation kit itself was tested in a negative control reaction containing pIVEX vector without any insert (lane: negative control). P and S denote pellet and supernatant, respectively.

We tested the concept of computational gene in an *in vitro *translation system. As structural gene of the computational gene we took the human inhibitor of DNA binding 1 (hID1) gene [34] comprised of two exons (452 bp and 115 bp) separated by one intron (239 bp). Its original intron sequence was modified to construct the initial and accepting final sites: the intron was split into two parts (by HindIII digestion), releasing intact exons and the upstream promoter region. The conserved splicing signals, i.e., 5'-splice signal, AG/GTRAG (995), CTSAY branch point (1213), strong poly(Y) signal between branch point and 3'-splice signal consisting of 13/14 pyrimidines (1218), and an NYAG/G 3'-splice signal (ACAG/G, 1232) were used as constants in the computational gene and remained unchanged. Two 16 nt long ssDNAs (overhang A, 5'-GGCCGCAATTCCAAAC-3', and overhang B, 5'-CAATTCCAAATGAGCT-3') were attached to the released free overhangs after enzyme digestion, leaving 12 nt free overhangs, complementary to the 24 nt long activated diagnostic signal AB' (5'-CATTTGGAATTGGTTTGGAATTGC-3'). Note that the ligation point between overhangs A and B is located precisely at the mismatched positions in the diagnostic signal *AB' *(Figure 2B). Addition of the mutated DNA and diagnostic complexes, whose diagnostic signal is complementary to the single stranded overhangs in the initial and final accepting state, led to successful self-assembly of the double stranded long fragment, resembling the expected size of the gene of interest [see Additional file 5]. A viable functional gene was assembled in an *in vitro *translation system (Figure 3). All the components, the diagnostic complex (*Am/AB'*), the mutated DNA (*Am'*), and the non-assembled gene fragments (Figure 2), were mixed together and added simultaneously to an eukaryotic *in vitro *translation system and the reaction proceeded for 3.5 h at the optimal conditions (for details see Methods section). The addition of mutated DNA (*Am' *single strand) initiated the programmed cascade reaction specified by the path: (i) mutation detection, (ii) gene self-assembly and linking, and (iii) transcription and translation, yielding a 155 amino acid long protein (17.3 kDa) as output. The size of the output was verified by a control reaction, containing the intact hID1 gene (Figure 3, positive control lane). Even thought the yield of the output is very low (weak band, Figure 3), it points out the potential for feasibility of the model. Without a released diagnostic signal no self-ligation of the therapeutic gene could be detected (Figure 3, self-ligation control). The *in vitro *translation system is representative of intact transcription/translation machinery in the cell and the results from this experiment suggest that successful self-assembly of the computational gene and translation into a viable product (protein or anti-drug) might occur in living cells.

If the promotor-containing fragment starts transcription before the gene self-assembly (hence leading to an incomplete mRNA transcript), the reliability of the computation would not be affected. In eukaryotic cells, quality control mechanisms ensure that an mRNA molecule is complete before translation is initiated. These mechanisms include cleavage and polyadenylation of the 3' end of proper RNA transcripts protecting them from cellular degradation [35].

### Generalisation

The diagnostic rule (1) can be generalised to n ≥ 1 disease-related mutations,

if proteinX_mutated_at_codon_Y1 and……and proteinX_mutated_at_codon_Ynthen produce_drug fi
 MathType@MTEF@5@5@+=feaafiart1ev1aaatCvAUfKttLearuWrP9MDH5MBPbIqV92AaeXatLxBI9gBaebbnrfifHhDYfgasaacH8akY=wiFfYdH8Gipec8Eeeu0xXdbba9frFj0=OqFfea0dXdd9vqai=hGuQ8kuc9pgc9s8qqaq=dirpe0xb9q8qiLsFr0=vr0=vr0dc8meaabaqaciaacaGaaeqabaqabeGadaaakeaafaqaaeGacaaabaGaeeyAaKMaeeOzayMaeeiiaaIaeeiCaaNaeeOCaiNaee4Ba8MaeeiDaqNaeeyzauMaeeyAaKMaeeOBa4gcbiGae8hwaGLaee4xa8LaeeyBa0MaeeyDauNaeeiDaqNaeeyyaeMaeeiDaqNaeeyzauMaeeizaqMaee4xa8LaeeyyaeMaeeiDaqNaee4xa8Laee4yamMaee4Ba8MaeeizaqMaee4Ba8MaeeOBa4Maee4xa8Lae8xwaK1aaSbaaSqaaGqaaiab+fdaXaqabaGccqqGGaaicqqGHbqycqqGUbGBcqqGKbazcqWIMaYsaeaacqWIMaYscqqGHbqycqqGUbGBcqqGKbazcqqGGaaicqqGWbaCcqqGYbGCcqqGVbWBcqqG0baDcqqGLbqzcqqGPbqAcqqGUbGBcqWFybawcqqGFbWxcqqGTbqBcqqG1bqDcqqG0baDcqqGHbqycqqG0baDcqqGLbqzcqqGKbazcqqGFbWxcqqGHbqycqqG0baDcqqGFbWxcqqGJbWycqqGVbWBcqqGKbazcqqGVbWBcqqGUbGBcqqGFbWxcqWFzbqwdaWgaaWcbaGae8NBa4gabeaaaOqaaiabbsha0jabbIgaOjabbwgaLjabb6gaUjabbccaGiabbchaWjabbkhaYjabb+gaVjabbsgaKjabbwha1jabbogaJjabbwgaLjabb+faFjabbsgaKjabbkhaYjabbwha1jabbEgaNjabbccaGiabbAgaMjabbMgaPbqaaaaaaaa@A165@

This rule can be realized by using an (*n *+ 2)-state *n*-symbol automaton (Figure 1C). In the (*i *- 1)-th state, the input symbol given by the *i*-th mutation lets the automaton transit into the *i*-th state. The automaton can be implemented by two partially dsDNA molecules, ssDNA molecules related one-to-one with the mutations, and further (complementary) ssDNA molecules necessary for self-assembly. The functional gene will be self-assembled only if the *n*-th (final) state is reached, that is, if all *n *diagnosed mutations are present [see Additional file 6]. In this way, computational genes may be used for the diagnosis and therapy of diseases related to mutation of transcribed genetic material. The rule (2) may even be generalised to involve mutations from different proteins allowing a combined diagnosis and therapy. Furthermore, computational genes are extendable to prokaryotic genes evidencing the generality of the principle. A prokaryotic model could release several different output molecules in response to different environmental conditions [see Additional file 7]. Diagnostic rule (2) can be implemented by linear self-assembly that was successfully demonstrated in the Adleman's first experiment [36], and recently has been used to implement *in vitro *finite small-state automata [37].

## Conclusion

Our work demonstrated the basic principles of computational genes and particularly, their potential to detect mutations, and as a response thereafter to administer an output that suppresses the aberrant disease phenotype and/or restores the lost physiological function. In this way, computational genes might allow implementation *in situ *of a therapy as soon as the cell starts developing defective material. Computational genes combine the techniques of gene therapy which allows to insert a healthy gene into the genome replacing an aberrant gene, as well as antisense technology mediating gene silencing. In addition, a computational gene can theoretically implement general *m*-state *n*-symbol automata and could be designed to solve all types of finite state applications at the molecular level, thus offering tremendous advantage for broader applicability than previous approaches [3-6], whose complexity is limited to fewer states and symbols. Clearly, *in vivo *application of computational genes is the ultimate goal, but before this some hurdles need to be overcome, i.e., the internalisation of the computational gene into the cell, its longevity, and its integrity in the cell. The issue of integrating computational genes into cellular regulatory pathways is directly linked to other biomolecular computing approaches in which gene regulation is the basis for making computations [38]. Computational genes provide a major step towards bringing molecular computers to work inside living organisms, to detect and correct physiological abnormalities, and to administer healthy solutions.

## Methods

### DNA manipulations

The hID1 gene (pBLAST49-hID1, Invivogen) was subcloned from into the pIVEX 2.3d vector (Roche-Applied-Science). The resulting pIVEX-hID1 plasmid was mutated (Quickchange, Stratagene) using conservative replacements (no change in the amino acid sequence) to introduce the necessary restriction sites for generating initial and final accepting states and to modify both termini with *NotI *and *SacI *restriction sites [Additional file 2].

### *In vitro *translation

The plasmid pIVEX-hID1 was expressed in *in vitro *translation TNT T7 coupled reticulocyte lysate system (Promega) changing thereby the procedure provided by the manufacturer as follows: To the transcription/translation reaction mixture provided in the kit (total volume of 25*μ*l) 2*μ*l T7 RNA polymerase, 1*μ*l T4 DNA Ligase (New England Biolabs) and 2 *μ*l ^35^S-methionine (Amersham) were added. The self-assembly reaction contained 2*μ*l digested hID1, overhang A, overhang B, *Am/AB' *and *Am'*, pre-mixed in equimolar concentration and added to the final concentration of 0.4*μ*M to the *in vitro *translation reaction. The reaction for testing the self-ligation of the computational gene contained all the components as described for the self-assembly reaction, except for the *Am' *strand. The positive control reaction was supplied with 2*μ*l intact pIVEX-hID1 plasmid, and the negative control reaction contained only 2*μ*l pIVEX vector. The reactions were carried out at 30°C for 3.5 h. The translation product was resolved on 15% SDS-PAGE and detected by autoradiography.

### Strand exchange

The full sequences of the oligonucleotides *Am, Am'*, and *AB' *are included in Additional file 6. Equimolar amounts of *Am *and *AB' *dissolved in 150 *m*M sodium phosphate buffer pH 7.4 were denatured at 95°C for 30 min and slowly cooled down to 37°C with 1°C step to form stable dsDNA complexes. The displacement reaction was initiated by addition of the displacing strand *Am' *in different molar ratios to 1*μ*M *Am/AB' *duplex and was monitored at 260 nm [39]. The rate constant was determined by fitting the experimental data to a first-order reaction equation as described elsewhere [40,41]: (*A*_*t *_- *A*_0_) = (*A*_∞ _- *A*_0_)(1 - *e*^-*kt*^), where *t *is time, and *A*_*t*_, *A*_0_, and *A*_∞ _are the absorbance values at time *t*, zero and infinite time, respectively. The melting curves of the 1*μ*M dsDNA-duplexes *Am/Am' *and *Am/AB' *were monitored at 260 nm in phosphate buffer (see above) or in the presence of Ficoll 70 (150 g/L) mimicking the crowded environment in the cells [33].

## Competing interests

The authors declare competing interests (German patent Nr. 102006009000).

## Authors' contributions

IMMP developed the idea of computational genes, ZG performed the experiments, IMMP, ZI, and KHZ designed the experiments, discussed the results and wrote the manuscript. All authors read and approved the final manuscript.

## Supplementary Material

Additional file 1 A simple two-state one-symbol automaton implementing the rule (1). Click here for file

Additional file 2 Model for diagnosis and therapy of pathogenic mutations. A) Diagnostic process. A diagnostic complex is a dsDNA molecule resembling a short part of the functional gene of interest, in which one of the strands is intact (diagnostic signal) and the other bears the mutation to be detected (mutation signal). In case of a pathogenic mutation, the translated mRNA pairs to the mutation signal and triggers the release of the diagnostic signal. B) Therapy process. The released diagnostic signal completes the structure of the functional gene so that a wild-type protein or an anti-drug is provided by the transcription and translation machinery of the cell. Click here for file

Additional file 3 Displacement of the *Am *from the *Am/AB' *duplex. Different ratios of [*Am/AB'*]: [*Am'*] were tested as outlined in the figure legend. The oligonucleotides were dissolved in 150 *m*M sodium phosphate buffer (pH = 7.4) and added to 1*μ*M [*Am/AB'*] and the change in the absorbance at 260 nm was recorded at 37°C. Displacement curves are presented as normalized inverted values of the changes of the absorbance over the time. Click here for file

Additional file 4 Melting curves of mismatched (*Am/AB'*) and perfect (*Am/Am'*). The melting curves of *Am/AB' *and *Am/Am' *complexes were obtained by denaturing of 1*μ*M single stranded *Am *and *AB' *or *Am *and *Am' *at 95°C for 30 minutes, followed by a slow cool down to 37°C with 1°C step. The melting curves were monitored at 260 nm in 150 *m*M sodium phosphate buffer (pH = 7.4) (panel A) and in 150 *m*M sodium phosphate buffer (pH = 7.4) containing 150 g/L Ficoll as crowding agent (panel B). The melting curves in the presence of the crowding agent were monitored till 75°C due to increased bubbling of the solution at high temperatures and increased signal-to-noise ratio. Click here for file

Additional file 5 Verification of the computational gene constructs. Lane 1: Product A (926 bp). Lane 2: Product B (653 bp). Lane 3: Part B with ligated overhang B (final accepting state), followed by *HindIII *digestion. Lane 4: Part A with ligated overhang A (initial state), followed by *HindIII *digestion. Lane 5: complete functional gene (final acepting state, initial state, and *AB' *strand). The higher band appearing at 1.9 kb (lane 4) is presumably an AA-dimer. Click here for file

Additional file 6 A simple two-state two-symbol automaton implementing the rule (2), for *n*= 2. Click here for file

Additional file 7 Prokaryotic computational gene. Due to the peculiar arrangement of prokaryotic genes in operons, a prokaryotic model could release several different output molecules in response to different environmental conditions, making even more complex computations possible. The key patterns of the operon are the constants of the computational gene (DNA-polymerase, operator-binding segments, and start and stop codons). Click here for file
