# Supplementary figures and images for: Computational genes: a tool for molecular diagnosis and therapy of aberrant mutational phenotype
Source: BMC Bioinformatics. 2007 Sep 28;8:365. doi: 10.1186/1471-2105-8-365 (PMC2175521; doi:10.1186/1471-2105-8-365)

## Additional File 1

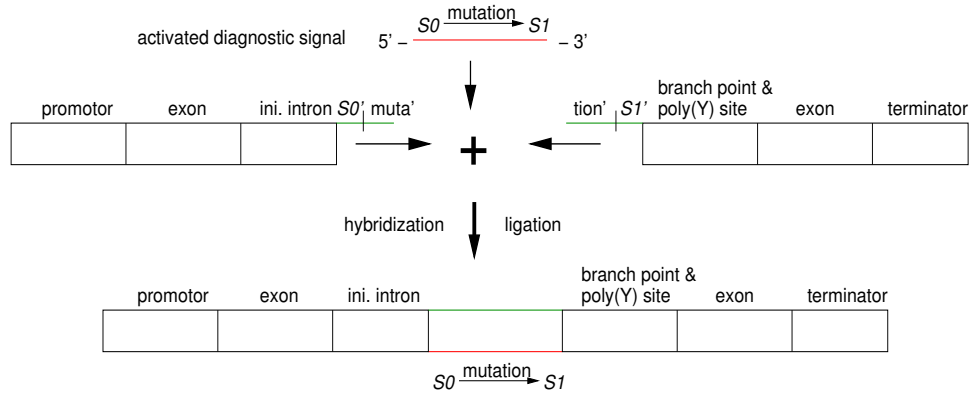

Figure 1: A simple two-state one-symbol automaton implementing the rule (1).

Supplement: Additional file 1 — A simple two-state one-symbol automaton implementing the rule (1). [file 1471-2105-8-365-S1.pdf]

## Additional File 6

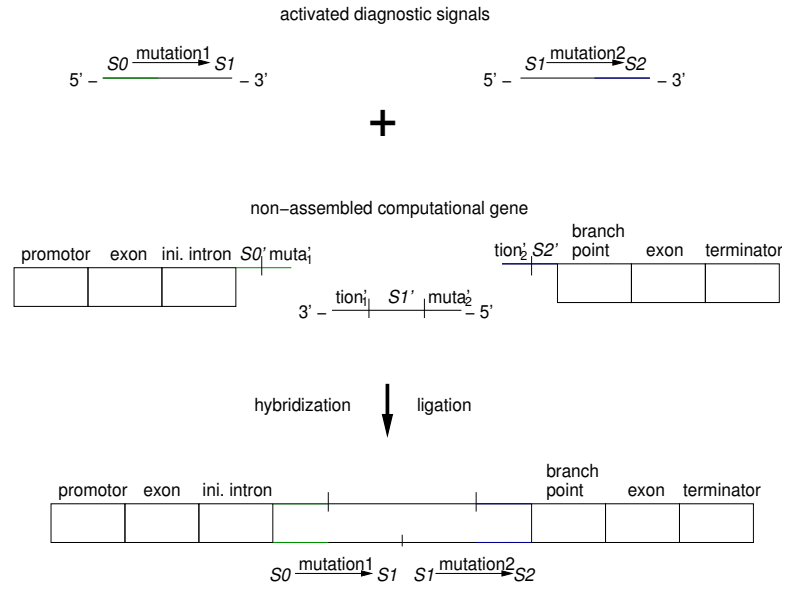

Figure 1: A simple two-state two-symbol automaton implementing the rule (2), for  $n = 2$ .

Supplement: Additional file 6 — A simple two-state two-symbol automaton implementing the rule (2), for n= 2. [file 1471-2105-8-365-S6.pdf]
