# Supplementary material for: Computational genes: a tool for molecular diagnosis and therapy of aberrant mutational phenotype
Source: BMC Bioinformatics. 2007 Sep 28;8:365. doi: 10.1186/1471-2105-8-365 (PMC2175521; doi:10.1186/1471-2105-8-365)
Supplement: Additional file 2 — Model for diagnosis and therapy of pathogenic mutations. A) Diagnostic process. A diagnostic complex is a dsDNA molecule resembling a short part of the functional gene of interest, in which one of the strands is intact (diagnostic signal) and the other bears the mutation to be detected (mutation signal). In case of a pathogenic mutation, the translated mRNA pairs to the mutation signal and triggers the release of the diagnostic signal. B) Therapy process. The released diagnostic signal completes the structure of the functional gene so that a wild-type protein or an anti-drug is provided by the transcription and translation machinery of the cell. [file 1471-2105-8-365-S2.pdf]

Additional File 2

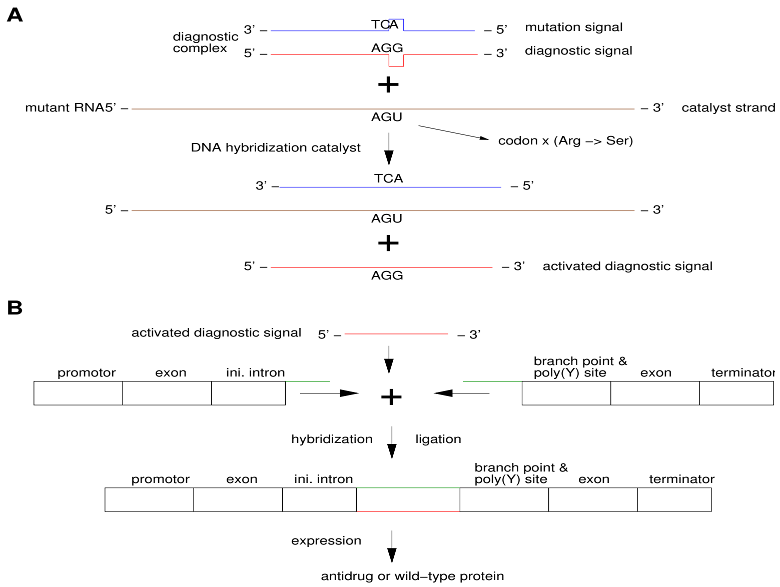

Figure 1: Model for diagnosis and therapy of pathogenic mutations (see 'Additional Files' Section in paper).
