# Supplementary material for: Computational genes: a tool for molecular diagnosis and therapy of aberrant mutational phenotype
Source: BMC Bioinformatics. 2007 Sep 28;8:365. doi: 10.1186/1471-2105-8-365 (PMC2175521; doi:10.1186/1471-2105-8-365)
Supplement: Additional file 3 — Displacement of the Am from the Am/AB' duplex. Different ratios of [Am/AB']: [Am'] were tested as outlined in the figure legend. The oligonucleotides were dissolved in 150 mM sodium phosphate buffer (pH = 7.4) and added to 1μM [Am/AB'] and the change in the absorbance at 260 nm was recorded at 37°C. Displacement curves are presented as normalized inverted values of the changes of the absorbance over the time. [file 1471-2105-8-365-S3.pdf]

Additional File 3

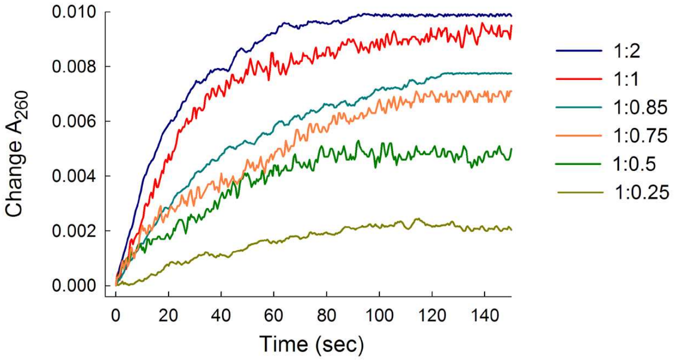

Figure 1: Displacement of the *Am* from the *Am/AB'* duplex. Different ratios of [*Am/AB'*]: [*Am'*] were tested as outlined in the figure legend. The oligonucleotides were dissolved in 150 *m*M sodium phosphate buffer (pH=7.4) and added to 1 μM [*Am/AB'*] and the change in the absorbance at 260 nm was recorded at 37°C. Displacement curves are presented as normalized inverted values of the changes of the absorbance over the time.
