# Supplementary material for: Computational genes: a tool for molecular diagnosis and therapy of aberrant mutational phenotype
Source: BMC Bioinformatics. 2007 Sep 28;8:365. doi: 10.1186/1471-2105-8-365 (PMC2175521; doi:10.1186/1471-2105-8-365)
Supplement: Additional file 4 — Melting curves of mismatched (Am/AB') and perfect (Am/Am'). The melting curves of Am/AB' and Am/Am' complexes were obtained by denaturing of 1μM single stranded Am and AB' or Am and Am' at 95°C for 30 minutes, followed by a slow cool down to 37°C with 1°C step. The melting curves were monitored at 260 nm in 150 mM sodium phosphate buffer (pH = 7.4) (panel A) and in 150 mM sodium phosphate buffer (pH = 7.4) containing 150 g/L Ficoll as crowding agent (panel B). The melting curves in the presence of the crowding agent were monitored till 75°C due to increased bubbling of the solution at high temperatures and increased signal-to-noise ratio. [file 1471-2105-8-365-S4.pdf]

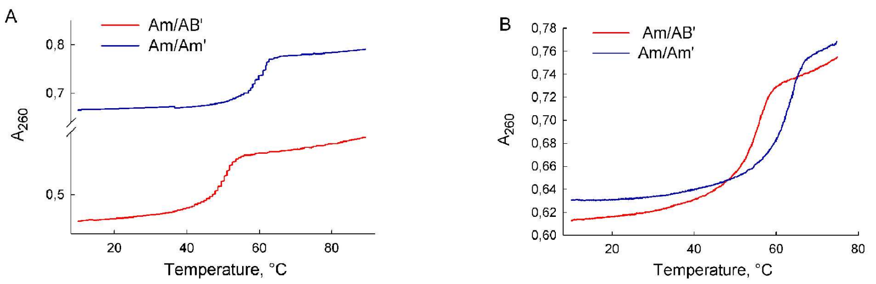

Figure 1: Melting curves of mismatched (*Am/AB'*) and perfect (*Am/Am'*). The melting curves of *Am/AB'* and *Am/Am'* complexes were obtained by denaturing of 1 μM single stranded *Am* and *AB'* or *Am* and *Am'* at 95°C for 30 minutes, followed by a slow cool down to 37°C with 1°C step. The melting curves were monitored at 260 nm in 150 *m*M sodium phosphate buffer (pH=7.4) (panel A) and in 150 *m*M sodium phosphate buffer (pH=7.4) containing 150 g/L Ficoll as crowding agent (panel B). The melting curves in the presence of the crowding agent were monitored till 75°C due to increased bubbling of the solution at high temperatures and increased signal-to-noise ratio.

Additional File 4
