# Supplementary material for: Computational genes: a tool for molecular diagnosis and therapy of aberrant mutational phenotype
Source: BMC Bioinformatics. 2007 Sep 28;8:365. doi: 10.1186/1471-2105-8-365 (PMC2175521; doi:10.1186/1471-2105-8-365)
Supplement: Additional file 5 — Verification of the computational gene constructs. Lane 1: Product A (926 bp). Lane 2: Product B (653 bp). Lane 3: Part B with ligated overhang B (final accepting state), followed by HindIII digestion. Lane 4: Part A with ligated overhang A (initial state), followed by HindIII digestion. Lane 5: complete functional gene (final acepting state, initial state, and AB' strand). The higher band appearing at 1.9 kb (lane 4) is presumably an AA-dimer. [file 1471-2105-8-365-S5.pdf]

Additional File 5

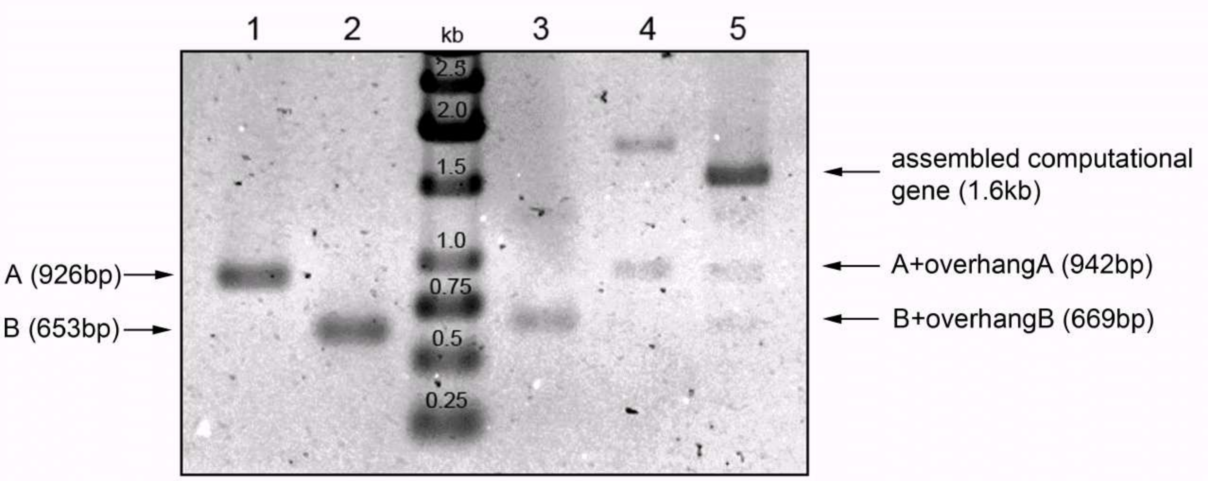

Figure 1: Verification of the computational gene constructs. Lane 1: Product A (926 bp). Lane 2: Product B (653 bp). Lane 3: Part B with ligated overhang B (final accepting state), followed by *HindIII* digestion. Lane 4: Part A with ligated overhang A (initial state), followed by *HindIII* digestion. Lane 5: complete functional gene (final acepting state, initial state, and *AB'* strand). The higher band appearing at 1.9 kb (lane 4) is presumably an AA-dimer.
