# Supplementary material for: Computational genes: a tool for molecular diagnosis and therapy of aberrant mutational phenotype
Source: BMC Bioinformatics. 2007 Sep 28;8:365. doi: 10.1186/1471-2105-8-365 (PMC2175521; doi:10.1186/1471-2105-8-365)
Supplement: Additional file 7 — Prokaryotic computational gene. Due to the peculiar arrangement of prokaryotic genes in operons, a prokaryotic model could release several different output molecules in response to different environmental conditions, making even more complex computations possible. The key patterns of the operon are the constants of the computational gene (DNA-polymerase, operator-binding segments, and start and stop codons). [file 1471-2105-8-365-S7.pdf]

Additional File 7

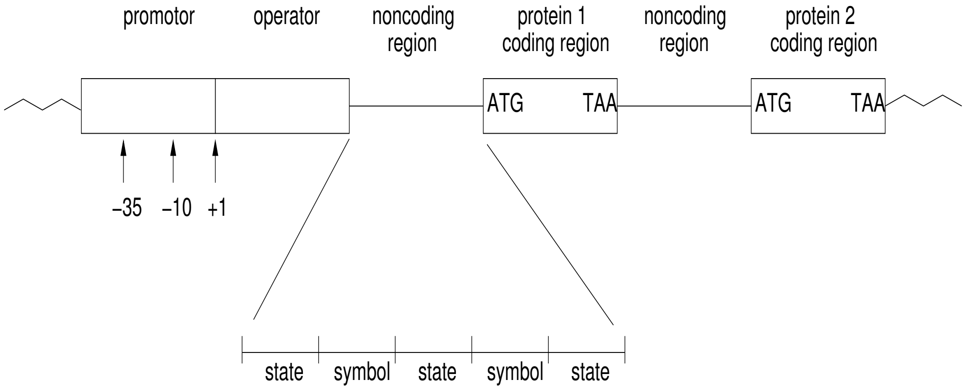

Figure 1: Prokaryotic computational gene. Due to the peculiar arrangement of prokaryotic genes in operons, a prokaryotic model could release several different output molecules in response to different environmental conditions, making even more complex computations possible. The key patterns of the operon are the constants of the computational gene (DNA-polymerase, operator-binding segments, and start and stop codons).
